# Combination of bioelectrochemical systems and electrochemical capacitors: Principles, analysis and opportunities

**DOI:** 10.1016/j.biotechadv.2019.107456

**Published:** 2020

**Authors:** Leire Caizán-Juanarena, Casper Borsje, Tom Sleutels, Doekle Yntema, Carlo Santoro, Ioannis Ieropoulos, Francesca Soavi, Annemiek ter Heijne

**Affiliations:** a Environmental Technology, Wageningen University, P.O. Box 17, Bornse Weilanden 9, Wageningen 6708 WG, the Netherlands; b Wetsus, European Centre of Excellence for Sustainable Water Technology, Oostergoweg 9, Leeuwarden 8911MA, the Netherlands; c Bristol BioEnergy Centre, Bristol Robotics Laboratory, T-Block, UWE, Coldharbour Lane, Bristol BS16 1QY, United Kingdom; d Department of Chemistry “Giacomo Ciamician”, Alma Mater Studiorum Università di Bologna, Via Selmi 2, Bologna 40126, Italy

**Keywords:** Microbial fuel cell, Capacitance, Electrical double-layer, Scaling up, Supercapacitor, Power output

## Abstract

Bioelectrochemical systems combine electrodes and reactions driven by microorganisms for many different applications. The conversion of organic material in wastewater into electricity occurs in microbial fuel cells (MFCs). The power densities produced by MFCs are still too low for application. One way of increasing their performance is to combine them with electrochemical capacitors, widely used for charge storage purposes. Capacitive MFCs, i.e. the combination of capacitors and MFCs, allow for energy harvesting and storage and have shown to result in improved power densities, which facilitates the up scaling and application of the technology. This manuscript summarizes the state-of-the-art of combining capacitors with MFCs, starting with the theory and working principle of electrochemical capacitors. We address how different electrochemical measurements can be used to determine (bio)electrochemical capacitance and show how the measurement data can be interpreted. In addition, we present examples of the combination of electrochemical capacitors, both internal and external, that have been used to enhance MFC performance. Finally, we discuss the most promising applications and the main existing challenges for capacitive MFCs.

## 1 Introduction

### 1.1 Bioelectrochemical systems for current generation

Bioelectrochemical systems (BESs) employ microorganisms that catalyse an electrochemical reaction either at the anode, cathode or both. The first BES and, particularly, a microbial fuel cell (MFC) is attributed to M.C. Potter, who for the first time showed the consumption of organics by bacteria with the simultaneous production of electricity (Potter, 1911). The research into BESs has exploded since the discovery of microorganisms capable of direct extracellular electron transfer that was firstly reported in 1999 (Kim et al., 1999a, Kim et al., 1999b). The electron transfer mechanism itself, being either direct (via cytochromes or conductive extracellular structures) or indirect (via excreted or added mediators/electron shuttles), has led to numerous publications (Busalmen et al., 2008; Kracke et al., 2015; Lovley, 2006; Nielsen et al., 2009; Patil et al., 2012; Reguera et al., 2005, Reguera et al., 2006; Schröder, 2007; Xiu et al., 2019; Liu et al., 2016; Li et al., 2018). Additionally, application of BESs has grown in a variety of fields, such as wastewater treatment, bioremediation, desalination, recovery of nutrients, and biosensors (Kelly and He, 2014; Nancharaiah et al., 2015; Pant et al., 2010; Rabaey, 2009; Rodríguez-Arredondo et al., 2015). Several reviews have already focused on organic carbon (Pant et al., 2010), nitrogen (Kelly and He, 2014; Rodríguez-Arredondo et al., 2015), sulphur (Rabaey, 2009) and metal (Nancharaiah et al., 2015), as possible electron donors and acceptors in BESs. Regarding the conversion of organic waste into electricity, the primary objective is to efficiently produce current from the available substrate (Pham et al., 2009). Highest current densities are produced in anodes where most of the available biomass is attached to the electrode and not suspended as planktonic biomass (Franks et al., 2010; Khan et al., 2016). The combination of biofilm and electrode is generally referred to as a bioanode, which is the responsible electrode for substrate oxidation. On the cathode, protons and electrons converge and a (bio)electrochemical reduction reaction occurs. In the case of MFCs, a reactant, usually oxygen, is reduced to water or hydroxide ions depending on the electrolyte pH (Kinoshita, 1992; Slate et al., 2019). In the case of microbial electrolysis cells (MECs), hydrogen or methane is produced as final product through an external applied voltage (Call and Logan, 2008; Van Eerten-Jansen et al., 2012).

The performance of an MFC is generally determined by the Coulombic Efficiency (CE), cell voltage and current. The CE describes which part of electrons from the substrate end up in the electrical circuit. It is lower than 100% when competing (microbial) conversions take place, such as methanogenesis or sulphate reduction, or when electrons are consumed for biomass formation (Sleutels et al., 2011). Therefore, the CE has an effect on the energy efficiency of the system. This energy efficiency is also affected by the cell voltage, which in practise is a function of current. The thermodynamic cell voltage at neutral pH is ≈1100 mV, which is the difference between cathode and anode equilibrium potentials. The anode equilibrium potential is −496 mV (vs Ag/AgCl 3 M KCl) for the acetate/CO_2_ redox couple ([HCO_3_^−^] = 5 mM, [CH_3_COO^−^] = 5 mM, pH = 7), and the cathode equilibrium potential is +605 mV (vs Ag/AgCl 3 M KCl) for oxygen reduction (pO_2_ = 0.2, pH = 7) (Logan et al., 2006).

At open circuit conditions, when no current is flowing, the measured cell voltage should match the thermodynamic cell voltage. Under fully anaerobic conditions, in the presence of a sufficiently high acetate concentration and with a well-developed electroactive biofilm, the anode open circuit potential (OCP) approaches the thermodynamic one. On the contrary, in neutral media, the cathode OCP can be significantly lower than the thermodynamic one for the redox couple O_2_/H_2_O (at pH= 7). Platinum has shown a strong oxygen reduction reaction (ORR) catalysis activity and has been largely used in the past. However, over time platinum has been replaced by alternative catalysts (e.g. platinum alloys, transition metal oxides) due to its high cost and low stability with components present in real wastewater (Santoro et al., 2019a; Wang et al., 2014a). Usually, the cathode potential is 300 mV lower when platinum or iron-based catalysts are used, and becomes even lower (400–500 mV) with carbonaceous metal-free catalysts (Kodali et al., 2017; Santoro et al., 2016b).

When the external circuit is closed and the MFC is connected to an external load, the actual cell voltage becomes lower than the open circuit voltage. The cell voltage decreases because part of the energy is dissipated in resistive contributions of the electrochemical cell components and processes, i.e. the internal resistances (Chen et al., 2019; Fan et al., 2008). The internal resistances include (i) the electronic resistance of current collectors and electrode materials, (ii) the ionic resistance that depends on wastewater conductivity, (iii) the membrane resistance (if applicable), and (iv) the charge transfer resistances that are directly related to the kinetics of redox reactions. Electrode kinetics include the activation overpotential related to electron transfer, and the mass transport losses related to diffusion, depletion or accumulation of the chemical species involved in the reactions (Bard and Faulkner, 2001; Jeremiasse et al., 2009). At the bioanode, potential losses due to internal resistances occur because of the complex nature of the anode electron transfer mechanisms, which are extensively discussed in literature (Kumar et al., 2017; Patil et al., 2012; Schröder, 2007). At the cathode, potential losses occur for the sluggish kinetics of the ORR. As the ORR at the cathode takes place in neutral media, the concentrations of both H^+^ and OH^−^, reagents for the ORR, are at the lowest value and lead to diffusion limitations. Many researchers aim to reduce the losses related to oxygen reduction, utilizing low-cost, environmentally friendly and durable (biological) catalysts, as described above (Kinoshita, 1992; Santoro et al., 2016a, Santoro et al., 2016b).

### 1.2 Limited power density for commercialization of MFCs

For MFCs to become competitive with mature and commercially available wastewater treatment technologies like anaerobic digestion, a power density of 1000 W m^−3^ (reactor volume) would be required (Arends and Verstraete, 2012; ter Heijne et al., 2011). Power densities of MFCs have shown a stagnating trend over the last years (Logan et al., 2015), with a maximum value of 200 W m^−3^ of reactor volume or 2–3 W m^−2^ of projected membrane (or cathode) surface area. It is important to note that reported power densities are generally obtained in lab-scale systems, mostly under optimized conditions, with model substrates and considerable amounts of added salts and buffer and at high operating temperatures (Fan et al., 2008; Yang and Logan, 2016). Especially for application in wastewater treatment, electrolyte conductivity is low, pH is not controlled, temperature varies significantly being generally lower than in lab-scale experiments, and system clogging is a risk. All these factors reduce the power density compared to optimized conditions. Moreover, when scaling up MFCs, the limitations encountered at lab-scale become more prominent and additional limitations appear, such as energy losses due to pressure drop, increase of contact resistance, system mechanical integrity and use of highly conductive and thus expensive materials (Dekker et al., 2009; Heidrich et al., 2013; Rodenas Motos et al., 2017; Rossi et al., 2019; Zamora et al., 2017).

An alternative for scaling up MFCs, well known from chemical fuel cells and battery applications, is through the stacking of different cells that can be connected in series or in parallel (Aelterman et al., 2006; Ieropoulos et al., 2008; Liu et al., 2008; Shin et al., 2006). This configuration increases the complexity of control and equipment required to operate the overall system. In fact, a risk of stacking cell pairs is the occurrence of cell reversal, which decreases the performance of the full stack (Oh and Logan, 2007). Another approach for scaling up is to increase the number of separate modules used (Ieropoulos et al., 2013; Kim et al., 2010), which may take away some of the above mentioned disadvantages of larger-scale operation. However, separate modules often require more materials (connections, tubing, feed troughs) than stacked cells.

### 1.3 Exploiting electrochemical capacitive properties in BESs

Recently, it was demonstrated that the use of materials with electrochemical capacitive properties can enhance the power density of MFCs (Feng et al., 2014; Houghton et al., 2016; Lv et al., 2012, Lv et al., 2014; Santoro et al., 2017; Soavi et al., 2016). Capacitive materials possess a high specific surface area that allows for charge storage with the formation of an electrical double-layer (EDL). The use of capacitive materials was first explored for the so-called “biosupercapacitors”, which combined capacitive materials with enzymatic fuel cells (Agnès et al., 2014; Pankratov et al., 2014a, Pankratov et al., 2014b, Pankratov et al., 2016). Already in these early studies, increased current and power densities of these enzymatic fuel cells were reported compared to the use of non-capacitive materials.

The use of capacitors in combination with BESs has two advantages: (i) enhanced power density and (ii) flexibility of operation (Deeke et al., 2015; Wang et al., 2015). The enhanced power density is the result of the high specific surface area of electrodes, which decreases the local current density and thus overpotentials, and increases the overall current density. Several studies have shown that intermittent operation mode of an MFC can lead to an increased power density compared to the use of a continuous external load (Deeke et al., 2012; Dewan et al., 2009; Walter et al., 2014). In intermittent mode, current is delivered at high rate only for short periods (lower than minutes) thanks to the high charge density (and counter ions) stored within the EDL. This operation results in higher power densities compared to continuous operation mode. The flexibility of operation relates to the fact that capacitive BESs can buffer discontinuities of current in the small-time scale and deliver on-demand energy. This feature is extremely useful in the context of energy storage of renewable energy, which usually cannot provide a constant power generation. Due to this flexible and dynamic electrochemical response, capacitive BESs can be combined with batteries or other energy harvesters (e.g. fuel cells, solar cells) (Wang et al., 2015).

**Fig. 1:**
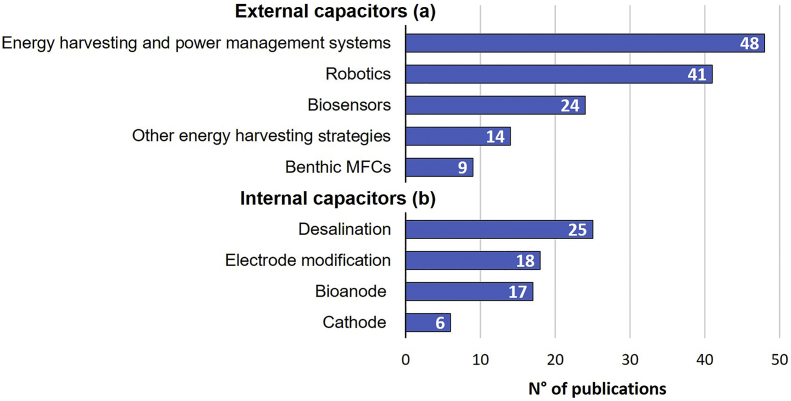
Number of publications, ordered from high to low, related to the use of capacitors in combination with BES. Publications are classified based on the type of capacitor involved, i.e. (a) external (outside in the circuit) or (b) internal (inside the cell). Search results from Scopus with search terms “microbial fuel cell” in combination with the following terms: “robotics”, “capacitive desalination”, “sensor and capacitor”, “power management strategies”, “power management system and capacitor”, “power management system and charge pump”, “power management system and boost converter”, “power management system and maximum power point”, “energy harvesting system and capacitor”. In addition, the search term “microbial capacitive deionization” was used. All publications are dated between 2000 and 2019.

So far, over 200 papers have been published describing the combined use of capacitors and BESs. Fig. 1 shows the most relevant applications of BES that use capacitors either in the external circuit (e.g. robotics (Ieropoulos et al., 2012; Walters et al., 2013), biosensor (Liu et al., 2015; Zhang et al., 2011), benthic MFCs (Arias-Thode et al., 2017; Tender et al., 2008)) or integrated in the electrochemical cell (e.g. desalination (Meng et al., 2017; Stoll et al., 2015)). The study of power management systems and energy harvesting systems to optimize the power output of MFCs, which usually include a capacitor, is also extensive (including electronic circuits with all kind of transformers, converters and boosters) (Erbay et al., 2014; Do Park and Ren, 2012a). Other energy harvesting strategies include the use of external capacitors to e.g. avoid voltage reversal (Papaharalabos et al., 2017), the use of intermittent energy harvesting operation mode (Walter et al., 2014) or the optimization of external resistances to enhance cell performance (Pinto et al., 2011). As for internal capacitors, many studies focus on the modification of electrode materials to develop or increase their capacitive properties in order to integrate them in BESs (Chen et al., 2018; Feng et al., 2014). Similarly, the application of capacitive materials as electrodes is extensively used in MFCs mostly in the anode (Wang et al., 2016, Wang et al., 2018) but also in the cathode (Ansari et al., 2016; Santoro et al., 2017).

When internal capacitors are used in MFCs, they exploit inherent or additional capacitive features of the anode and/or cathode. In this manuscript, we discuss the use of electrochemical capacitors and capacitive electrodes in BESs with focus on power production in MFCs. First, an overview of the theory on electrochemical capacitors is provided (Section 2), followed by how capacitance can be measured with the use of different electrochemical techniques (Section 3). After, the integrated use of electrochemical capacitors in MFCs, both in anodes and cathodes, is explained, with focus on capacitive materials and examples of applications (Section 4). The use of external capacitors is also addressed, where their behaviour under cell intermittent operation mode and their use on the research field of robotics is explained (Section 5). A future outlook on the combination of BESs and electrochemical capacitors is finally presented (Section 6).

## 2 The electrical double-layer and BES

The capacitance of a material reflects the ability to store charge, and thus understanding of capacitance is crucial to get insight on how such materials can be combined with MFCs. The value of capacitance (Eq. (1)), expressed in Farad (F), corresponds to the amount of charge (Q) that can be stored over a potential difference (∆V) of 1 V, and for ideal systems is a constant, i.e.:

(1) $$C=\frac{\mathrm{dQ}}{\mathrm{dV}}$$

**Fig. 2:**
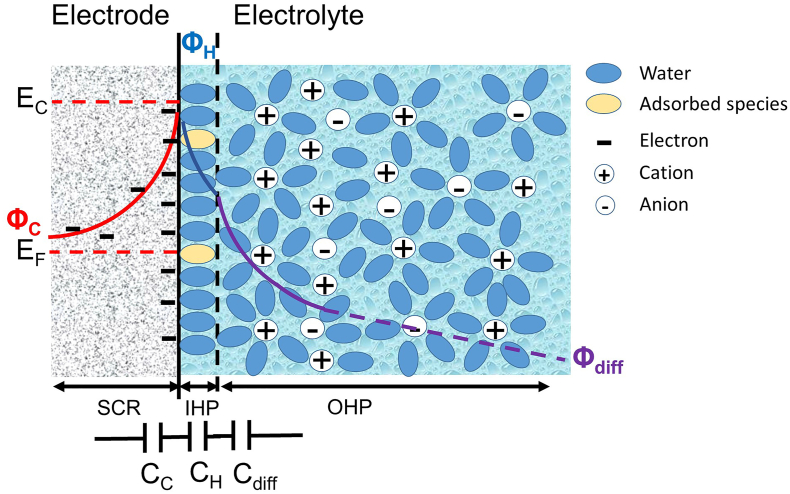
Scheme of the electrical double-layer at the solid-electrode/electrolyte interface with the formation of the space-charge region (SCR) in the solid electrode, the Inner Helmholtz Plane (IHP) and diffuse layer (Outer Helmholtz Plane, OHP) in the electrolyte. For electrolyte concentrations higher than 10^−2^ M, the typical total thickness of IHP and OHP is ca. 10 nm (Bard and Faulkner, 2001). The potential trend within the three regions (Φ_C_, Φ_H_, Φ_diff_) and the equivalent circuit that models the three capacitive components (C_C_, C_H_, C_diff_) of the interfaces are reported. E_C_ and E_F_ are the electrode conduction and Fermi level, respectively. The Fermi level represents the average energy of available electrons.

When a porous electrode is polarized, charge carriers can distribute into the bulk of the electrode over a relatively large distance (screening length) that is inversely related to the charge-carrier density; such region is called space-charge region (SCR) and ranges between a few angstroms to several thousands of angstroms in semiconductors but it is negligible in metals (Bard and Faulkner, 2001). On the electrolyte side, the formation of a compact layer of ions of the same charge (but different sign with respect to the electrode surface), the Inner Helmholtz Plane (IHP), forms at the closest distance from the electrode while a diffuse layer, he Outer Helmholtz Plane (OHP), forms at largest distance from the electrode (see Fig. 2).

With the formation of an EDL in the solid part of the interface and in the electrolyte, charge is distributed and potential gradients develop in the SCR, IHP and OHP regions. These three potential gradients (Φ_C_, Φ_H_ and Φ_diff_) make up three capacitive components (C_C_, C_H_ and C_diff_) that are connected in series and contribute to the electrode double-layer capacitance (C_dl_) as shown in Eq. (2):

(2) $$\frac{1\;}{C_{\mathrm{dl}}}=\frac{1\;}{C_{C}}+\frac{1\;}{C_{H}}+\frac{1\;}{C_{\mathrm{diff}}}$$

where C_c_ is the SCR capacitance, C_H_ is the capacitance related to the compact layer and C_diff_ is the capacitance of the diffuse layer. Hence, C_dl_ will be determined by the smallest of the capacitive components. In the case of EDL capacitors (EDLCs), high conductive electrodes and concentrated solutions are used, hence C_C_ and C_diff_ are high and EDL formation is only driven by C_H_.

According to the Helmholtz model, for concentrated solutions (typically 1 M for EDLCs) the capacitance C_H_ at each electrode interface is related to the surface area of the electrode (A) as stated in Eq. (3):

(3) $$C_{H}=\frac{k_{0}\;\varepsilon \;A}{\delta_{\mathrm{dl}}}$$

where k_0_ is the vacuum permittivity (8.85 10^−12^ F m^−1^), ε is the dielectric constant of the EDL region (that depends on solvent chemistry), and δ_dl_ is the thickness of the double-layer (δ_dl_; in the order of 10^−10^ m).

Carbon electrodes that have up to 2000 m^2^ g^−1^ of specific surface area (A), have a specific double-layer capacitance in the order of 100–150 F g^−1^. Pore size and distribution of the capacitive electrode plays an important role in the formation of EDL. An optimal combination of micropores (<2 nm), mesopores (2–50 nm) and macropores (>50 nm) in the electrode structure and a good connection network between them will minimize transport resistances of ions and maximize formation of EDL. In the same way, ionic composition and concentration of the electrolyte are important parameters for the formation (Béguin et al., 2014; Conway, 1999; Frackowiak and Béguin, 2001).

Materials that feature fast and reversible redox processes and exhibit a linear dependence of the charge stored with the potential can be termed pseudocapacitive electrodes (Brousse et al., 2015). In pseudocapacitive and other redox materials (i.e. materials that undergo faradic reactions but that cannot be termed pseudocapacitive), the faradic processes involve the bulk material and not only the surface. Therefore, charge accumulation at the IHP is higher with respect to EDLCs carbon electrodes, which increases the capacity and energy storage capability of the cells.

The maximum practical cell voltage (V_max_), together with the capacitance (C) and the internal resistance (i.e. the equivalent series resistance, ESR) of the capacitor, will determine the performance of EDLCs that can be analysed in terms of energy and power densities. Total capacitance results from the series combination of the two electrode capacitances as shown in Eq. (4):

(4) $$C={\left(\frac{1}{C_{\text{negative electrode}}}+\frac{1}{C_{\text{positive electrode}}}\right)}^{-1}$$

where C_negative electrode_ and C_positive electrode_ are the capacitance values of the negative and positive electrodes, respectively.

Therefore, in order to achieve high cell capacitance, both electrodes have to feature high capacitance values. In case of unbalanced values of electrode capacitances, the cell response will be driven by the least capacitive electrode. High specific energy of EDLCs (Eq. (5)) is achievable by high values of C and V_max_, which are dependent on the carbon electrode porosity and nanostructure and the electrochemical stability window of the electrolyte, respectively (Béguin et al., 2014; Conway, 1999). The maximum specific energy E_max_ can be calculated as follows:

(5) $$E_{\max}=\frac{1}{2}\frac{C\;{\left(V_{\max}\right)}^{2}}{m_{\mathrm{sc}}}$$

where m_sc_ is the total electrode mass.

High specific power (Eq. (6)) is achieved at low ESR, which in turn depends on: i) the conductivity of the electrolyte; ii) the electronic resistance of electrode materials; iii) the interfacial resistance between the electrode and the current collector; and iv) the ionic resistance of ions migrating/diffusing through the small pores of porous architectures of the electrode. The maximum specific power P_max_ is determined as follows:

(6) $$P_{\max}=\frac{1}{4}\;\frac{{\left(V_{\max}\right)}^{2}}{\mathrm{ESR}\;m_{\mathrm{sc}}}$$

The best performing commercially available EDLCs operate in organic electrolytes and feature E_max_ < 5 Wh kg^−1^, P_max_ < 10 kW kg^−1^ and V_max_ < 2.7 V (Béguin et al., 2014).

**Fig. 3:**
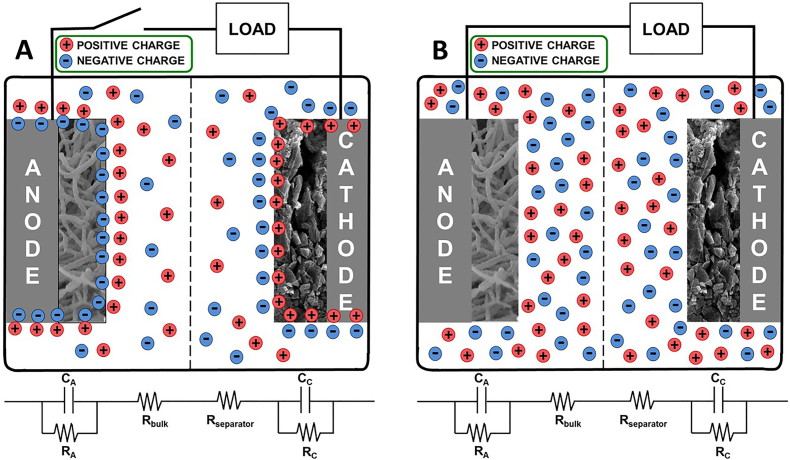
An MFC with a capacitive bioanode and a capacitive cathode. A) Charge storage in form of electrical double-layer in each of the electrodes at open circuit. B) Charge release from the electrode/electrolyte interface to the electrolyte at closed circuit. C_A_ and C_C_ refer to anode and cathode capacitances, respectively, and R_A_ and R_C_ refer to anode and cathode resistances, respectively. R_bulk_ refers to the resistance of the electrolyte and R_separator_ to the resistance of the membrane.

The integration of an EDLC with an MFC is the results of the combination of electrostatic (capacitive) and irreversible electrochemical (faradaic) processes that convert chemical energy into electrical energy. This concept notably differs from the working principle of hybrid, asymmetric or pseudocapacitors that work with reversible electrochemical processes. In most of the studies, the anode serves as a growth surface for electroactive bacteria, which release electrons to the anode via the bioelectrochemical oxidation of a substrate. In open circuit, the accumulation of electrons is responsible for the EDL formation at the anode/wastewater interface, where the surface negative charges are balanced by counter ions (cations) in the wastewater. The same process, but with opposite polarity, takes place at the cathode. This is charged positively due to reduction reactions (biological or chemical) occurring at the electrode, and balanced out by ions (anions) naturally occurring in the wastewater. Therefore, in equilibrium, the two electrodes work like the negative (anode) and positive (cathode) electrodes of an electrochemical capacitor that stores charge and energy by electrostatic charge separation at the two electrode EDLs (Fig. 3A). When the circuit of the MFC is closed, charges accumulated at the interface of both electrodes are been released to the electrolyte (Fig. 3B).

**Fig. 4:**
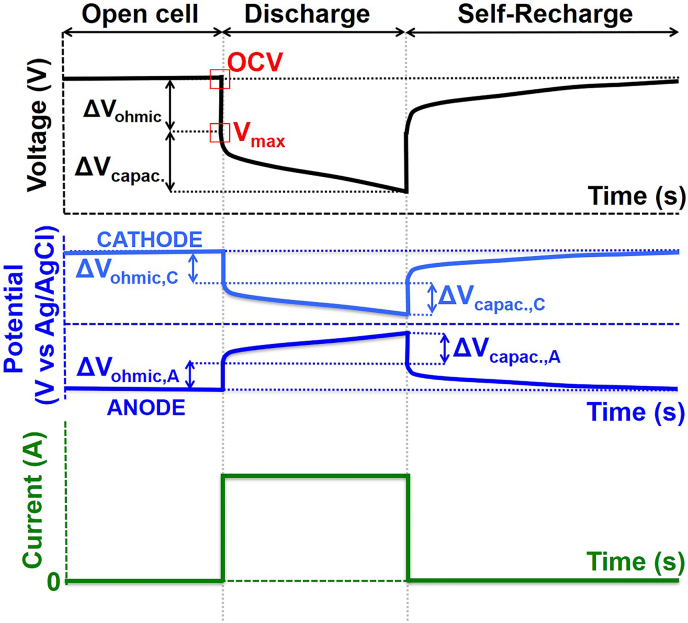
Cell voltage (top) and current (bottom) responses of a fully capacitive MFC (with biofilm on the anode) during a charge/discharge cycle after a period of open circuit. In the middle, the potential response of the cathode and anode electrodes. ∆V refers to the change in voltage that relates to two processes: the ohmic drop (∆V_ohmic_) and the change due to capacitance (∆V_capac._).

Fig. 4 shows the response of current and voltage of an EDLC-MFC during one rest (Open Cell) / galvanostatic discharge / self-recharge cycle. The responses of anode and cathode potentials are also shown. At open circuit, there is no current flowing through an external load, and both electrodes are polarized to a certain potential value resulting in a specific open circuit voltage (OCV).

When closing the circuit, the discharge occurs: the capacitive bioanode will simultaneously deliver the EDL stored charge (capacitive current) and the charge produced by the electroactive bacteria (faradaic current). At the same time, the oxygen cathode will simultaneously deliver the EDL stored charge and the charge produced by the oxygen reduction reaction. The anode potential will gradually increase (release of negative charges), while the cathode potential will decrease (release of positive charges). The initial change in potential consists of both the ohmic drop and the capacitance of the electrode (under fast discharge current regime and at short times). As a result of the changes in anode and cathode potentials, the cell voltage will continue decreasing during discharge. While the open circuit voltage (OCV) refers to the voltage at open circuit conditions, the useful cell voltage for power output will be that after the ohmic drop, referred to as V_max_.

In the rest step, the anode and cathode potentials will be restored to their original equilibrium values, i.e. the equilibrium potential of the electroacive bacteria in the anaerobic environment and the equilibrium potential of the oxygen cathode. This will result in an increase of cell voltage until again reaching the OCV, therefore this appears like a "self-recharge" of the EDL of the MFC.

Alternatively to internal EDLCs, external capacitors can also be connected to MFCs (see Section 5). Both for internal and external EDLCs, the evaluation of the capacitive response needs to consider the parallel faradaic processes taking place during the discharge of EDLC-MFC systems.

If no additional external load is connected, the current generated by the MFC (I_MFC_) charges the external EDLC up to a voltage that, in absence of leakage currents, corresponds to the highest voltage of the MFC exhibited in open circuit (OCV). The EDLC can then deliver the stored charge while being connected to the MFC (as internal capacitors), or instead disconnected (as in external capacitors). In the latter case, the discharge profile will be that characteristic of a conventional EDLC (and related to its ESR and C). For an EDL galvanosatic discharge at the set current I_EDLC_, the delivered charge is determined as in Eq (7):

(7) $$Q=I_{\text{EDLC}}\;\mathrm{dt}\;$$

When the EDLC is discharged while being connected with the MFC, the discharge behaviour will be different as the EDLC will deliver energy at the set current I_EDLC_ while being simultaneously recharged by the MFC (I_MFC_). The charge delivered by the EDLC-MFC when being connected is now determined as in Eq. (8):

(8) $$Q=I\;\mathrm{dt}=\left(I_{\mathrm{MFC}}+I_{\text{EDLC}}\right)\;\mathrm{dt}\;$$

and so the system features an apparent capacitance C′(Eq. (9)) higher than that exhibited by the EDLC alone and which can be calculated as follows:

(9) $$C\prime =\frac{\mathrm{dQ}}{\mathrm{dV}}=\left(I_{\mathrm{MFC}}+I_{\text{EDLC}}\right)\;\frac{\mathrm{dt}}{\mathrm{dV}}$$

The apparent capacitance concept applies even for other kinds of systems where an energy harvester is integrated with EDLC, as introduced by Intermite and co-workers (Intermite et al., 2017) for a solar cell-EDLC integrated device. Electrostatic and faradic processes have typically different rates and kinetics, with the latter being typically slower than the former. Therefore, it has to be expected that the apparent capacitive response of EDLC-MFC systems and/or of capacitive electrodes in MFCs might vary at different current regimes. While at high discharge currents and short times the cell response is mainly driven by the EDLC behaviour, at low currents and longer time the MFC redox processes mainly affect performance.

## 3 Measurement of capacitance using electrochemical techniques

Measurement of capacitance is crucial to determine how much charge can be stored by the electrode materials used in MFCs. Several electrochemical measurement techniques are available to determine the capacitance of electrodes and electrodes combined with microorganisms. When the test is performed in a 2-electrode mode, the overall cell response is evaluated and the capacitance measured is that of the cell. On the contrary, to evaluate the capacitance of a single electrode, the use of a reference electrode (3-electrode mode) is required.

**Fig. 5:**
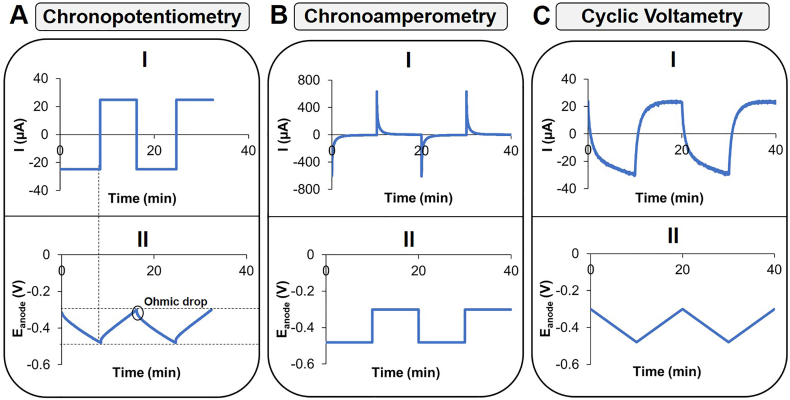
Current (I) and potential (II) responses of an abiotic capacitive anode (single activated carbon granule) during charge/discharge cycles (3 in total) with different electrochemical techniques: A) Chronopotentiometry; B) Chronoamperometry and C) Cyclic Voltammetry. Dotted lines (see A) in the x-axis mark the potential range at which the measurements were set, while dotted lines in the y-axis point the end of a charge/discharge cycle in both current and potential graphs. The black circle shows the ohmic (potential) drop when the cycle is changed.

In this section, the current-potential behaviour of a capacitive electrode is illustrated. The working electrode was a single activated carbon granule (weight = 1.03 mg, SSA = 764 m^2^ g^−1^), which was connected to a titanium wire as current collector (see (Caizán-Juanarena et al., 2019) for more information about the set-up). Control experiments with the current collector (without granule) showed that its contribution to current and capacitance was negligible. The reference electrode was Ag/AgCl (3 M KCl) and the counter electrode was graphite felt. The anolyte consisted of 50 mM phosphate buffer, a concentration that is relevant for BES, although different from that usually used during supercapacitor testing (often 0.1 or 1 M in acid or alkaline solutions). The cathode, separated from the anolyte by a cation exchange membrane (CEM), had 100 mM of [Fe(CN)_6_]^−3^ as electrolyte. Three electrochemical measurement techniques were used to measure the electrochemical response of this capacitive anode (Fig. 5): (i) chronopotentiometry; where current is controlled at negative and positive levels (also known as galvanostatic mode) (ii) chronoamperometry; where the electrode potential is controlled at fixed levels (also known as potentiostatic mode), and (iii) Cyclic Voltammetry (CV), where the electrode potential is changed linearly with time. All techniques were set up in a way that they followed a similar time response regime, meaning each cycle had a duration of about 10 min with similar scan rates and currents.

Fig. 5A shows the results obtained with chronopotentiometry measurements. At *t* = 0 min, a current of −25 μA is applied; electrons are transported into the granule and, as a result, the potential of the granule decreases to a (set) level of −0.48 V. When this occurs, at around *t* = 10 min, the current is changed to +25 μA and, as a result, electrons are transported from the granule to the counter electrode, leading to an increase of its potential (up to −0.3 V). The measured change in potential consists of two contributions: i) the ohmic potential drop that is related to the internal resistance and ii) the potential that is related to the capacitance. The ohmic drop can be seen in the graphs as an immediate steep change in potential (dV) when the current direction is altered. From that value, the electrode resistance, that includes the electrical resistances of the electrode and the electrolyte (the latter depends on the distance between the working electrode and reference electrode), can be calculated by the potential change divided by the momentary current change (R_electrode_ = dV/I). The total change in charge is calculated as in Eq. (7) andcapacitance as in Eq. (1).

Fig. 5B shows the results obtained with chronoamperometry measurements. At designated times (every 10 min in this case) a potential difference is applied between the granule and the reference electrode, which leads to transport of electrons into or out of the granule. While the potential values are steady, the current has a relatively high peak (I_p_) when the potential level changes, which is around 20 times higher than the current applied/achieved with the other two measurement techniques. In this way, the amplitude of the current is much higher (requiring a larger measurement range) and the current changes much faster, resulting often in a much lower measurement resolution that is more prone to errors even with higher data sampling rates. The potential difference divided by the peak current will give the value of the electrode resistance (I_p_ = V/R_electrode_).

Another way to calculate capacitance in this case is to use Eq. (10), which represents the current curve during chronoamperometry measurements.

(10) $$I=\frac{V}{R_{\mathrm{electrode}}\;}\;e^{-\frac{t}{\tau }}$$

where τ is the EDLC time constant, i.e. the time required for 63% of charge/discharge of the EDLC and t is the time (s). The capacitance can be derived by obtaining the value of τ from the measurement graph and dividing it by the measured electrode resistance (C_electrode_ = τ/R_electrode_).

Finally, Fig. 5C shows the results obtained with cyclic voltammetry measurements. To allow comparison with the other measurements, the cyclic voltammogram is split in such a way that current and potential responses are plotted separately as a function of time. At t = 0 min, the voltage is altered at a specific rate, in this case 0.3 mV s^−1^, so that the set potential range (−0.3 V to −0.48 V) is covered in 10 min. The potential changes linearly between these two limits, while the current changes fast right after one cycle and reaches a steady value towards the end of a cycle. The current is then directly proportional to the capacitance value and the rate of potential change (scan rate) (I=C × dV/dt). Here, the shape of the curve is also influenced by resistive components; it can be seen as the non-ideal box-shaped graph of the current. However, the value of electrode resistance is more difficult to extract from the graph.

**Fig. 6:**
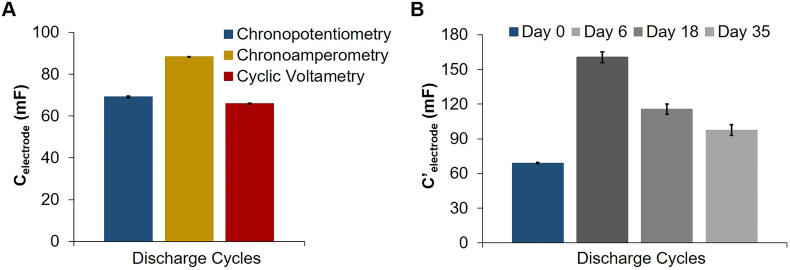
A) Capacitance of a single activated carbon (AC) granule calculated from the average of 3 discharge cycles with three different electrochemical techniques. B) Apparent capacitance of a single AC granule calculated with chronopotentiometry under abiotic (day 0) and biotic (days 6, 18 and 35) conditions.

From the discharge cycles (*n* = 3, although only two are shown), the electrode capacitance (Eq. (1)) was determined for each electrochemical technique and plotted in Fig. 6A. The highest capacitance was obtained for the chronoamperometry measurements (88 ± 0.05 mF), followed by chronopotentiometry (69.1 ± 0.5 mF), and cyclic voltammetry measurements (66 ± 0.2 mF). This shows that capacitance values have to be interpreted with care, as the measurement method used will influence the outcome. Fig. 6B shows the apparent capacitance of the same electrode, calculated with chronopotentiometry, in the presence of an electroactive biofilm at different growth stages: days 6, 18 and 35 after microbial inoculation. As explained in Section 2, when an electroactive biofilm is present also the current produced by the bacteria (I_MFC_) is measured together with the I_EDLC_ current (Eq. (8)), which results in an apparent capacitance (Eq. (9)). In the case of an electrode, its apparent capacitance also increases when an electroactive biofilm is present, in this case achieving 1.4 to 2.3 times higher values than the electrode capacitance under abiotic conditions (day 0). However, there was a decrease over time of the apparent capacitance, which could relate to a decrease of both the capacitive and/or redox performance of the bioanode as biofilm grows. On the one hand, micropores (<2 nm) and macropores (2–50 nm) are the main contributors to the high surface area of capacitive electrodes and thus EDL formation (Pandolfo and Hollenkamp, 2006), and even though these pores are inaccessible to bacterial cells (of 1 μm size, (Bond and Lovley, 2003)), they could block larger pores that act as path for ion transport during EDL formation. On the other hand, electroactive biofilms have shown to be limited in some processes (e.g. transport of nutrients and electron equivalents, movement of protons and pH-buffering compounds (Renslow et al., 2013)), so during the EDL formation process the transport of electron and protons, together with other counterions (Korth et al., 2015; Savéant, 1986), might influence charge storage capacity of the electrode when biofilm is present.

All above-mentioned measurements to determine the capacitance are based on direct current (DC), which are widely used and studied. However, increasingly often an alternating current (AC) technique, known as electrochemical impedance spectroscopy (EIS), is used. With EIS not only capacitance but also different processes that occur at the electrode/biofilm/solution interfaces (e.g. charge transfer, diffusion) can be distinguished (Dominguez-Benetton et al., 2012; Orazem et al., 2006; Orazem and Tribollet, 2008). There are several parameters to consider when performing EIS measurements, such as the electrode potential bias, AC voltage amplitude and range of frequencies (Yoho et al., 2015). Additionally, an equivalent circuit that fits the electrode material needs to be developed in order to obtain absolute values of capacitances and resistances. EIS is a powerful technique that permits to unravel the different contributes to the ohmic resistance and C_cell_ of the electrochemical cell as described in details in (Orazem and Tribollet, 2008).

Using EIS, it has been found that biofilms can store charge, which has been related to the c-type cytochromes that can accumulate charge (Malvankar et al., 2012). Ter Heijne and co-workers (ter Heijne et al., 2018) also calculated biofilm capacitance with EIS tests, with an increasing value during biofilm growth (up to 450 μF cm^−2^) and with a direct relation to current production. In this study, biofilm capacitance was determined using flat electrodes (Fluorinated Tin Oxide) with low surface area and thus capacitance (calculated as 25 μF cm^−2^). However, when capacitive electrodes are used to grow electroactive biofilm, differentiating between biofilm and electrode capacitances will again be difficult with AC techniques.

## 4 Integrated use of capacitors in MFCs

In this section, we will give an overview of the use of internal capacitors in MFCs by addressing the main existing research conducted in capacitive anodes and cathodes.

### 4.1 Integration of capacitive materials as anodes

The use of porous, three-dimensional electrodes for bioanodes is common, as they have a high surface area per volume ratio for biofilm growth that increases the bacteria/electrode interface. Electrode materials, ranging from carbonaceous materials (e.g. graphite fibers or plates, activated carbon granules, graphene, carbon cloth, carbon paper, carbon veil, carbon nanotubes) to metals like titanium, copper or stainless steel, have been a topic of extensive research with the aim of increasing bioanode performance (Baudler et al., 2015; Sleutels et al., 2011, Sleutels et al., 2009, Sleutels et al., 2017; ter Heijne et al., 2008; Wei et al., 2011; Zhou et al., 2011). Generally speaking, the available surface area for bioanode formation is a dominant factor in current production if substrate and internal resistance of the system are not limiting (Blanchet et al., 2016; Chong et al., 2018; Oliot et al., 2017; Wei et al., 2011). Providing sufficient area for microorganisms to have access to substrate and allow for adequate mixing to remove the produced protons is of utmost importance. The optimization of other electrode properties, e.g. surface chemistry (Guo et al., 2013; Teravest and Angenent, 2014) or biocompatibility (Guo et al., 2015), the improvement of cell configuration (Liu et al., 2005; Rodenas Motos et al., 2015) or the enhancement of electron transfer to and from the electrode (Ishii et al., 2018), are also well-known approaches to increase power densities by reducing internal resistances of BESs.

Operation of a capacitive anode with an electroactive biofilm in intermittent mode was first reported by Deeke and co-workers (Deeke et al., 2012). They made use of a layer of activated carbon that was casted on the surface of a current collector. The biofilm was grown on this capacitive electrode making up a capacitive bioanode. The relative charge recovery, i.e. charge transferred by the intermittent operation of the capacitive bioanode as compared to the continuous operation of a non-capacitive bioanode, was as high as 140% for a 0.2 mm capacitive layer. Thicker capacitive layers recovered overall lower charge, mostly with longer total charge/discharge cycle times, with values around 100% for a 0.5 mm layer and lower than 100% (down to 20%) for a 1.5 mm layer (Deeke et al., 2013). The differences in surface roughness and specific surface area confounded the results somewhat, however it was clear that the intermittent operation of this capacitive bioanode produced more overall charge compared to continuous operation of a non-capacitive graphite electrode.

#### 4.1.1 Activated carbon granules as bioanodes

Often, activated carbon (AC) is used as capacitive electrode material because it has a high specific surface area (up to 2000 m^2^ g^−1^). When used in granular form, it enables the separation of the charging and discharging processes in a fluidized capacitive reactor; granules are charged in one part of the reactor through oxidation of organic matter, and discharged at certain times by contacting the charged granules with a current collector to recover electricity (Borsje et al., 2019; Deeke et al., 2015). In Table 1 examples of reactor performances that use AC granules as electrode material in bioanodes are shown, with reactors ranging from mL to L scale. These reactors have been operated in intermittent mode either through intermittent control or through intermittent contact of the AC granules with the current collector (Borsje et al., 2016, Borsje et al., 2019; Deeke et al., 2015; Li et al., 2014; Liu et al., 2014a, Liu et al., 2014b; Tejedor-Sanz et al., 2017; Wang et al., 2014a).

**Table 1:** Performance overview of capacitive MFCs with activated carbon (AC) granules.

Reactor type	V_total_ (mL)	V_granules_ (mL)	Control mode	I_max_ (A m^−3^_reactor_) (I at P_max_)	I_max_ (A m^−3^_granule_) (I at P_max_)	Reference
Single granule fixed with wire current collector with intermittent control	1	0.01	−0.3 V vs Ag/AgCl	757	76,765	(Borsje et al., 2016)
Granules with intermittent contact to anode mesh via stirring	7	1.14	external resistor; air cathode	460 (260)	2837 (1603)	(Liu et al., 2014b)
Granules with intermittent contact to anode rod via fluidization through liquid pumping	40	2.27	−0.2 V vs Ag/AgCl	5	89	(Liu et al., 2014a)
Granules with intermittent contact to immersed anode plate via fluidization through liquid pumping	680	80	+0.2 V vs Ag/AgCl	25	214	(Tejedor-Sanz et al., 2017)
Granules with intermittent contact to tubular anode cloth via fluidization through liquid pumping	1000	300	48 Ω; air cathode	23 (11)	78 (37)	(Li et al., 2014)
Granules with intermittent contact to anode rod via fluidization through liquid pumping	1000	177	external resistor; air cathode	0.8 (0.4)	4.2 (2.3)	(Wang et al., 2014b)
Granules with intermittent contact to anode plate in external cell via transport using gas lift	2102	392	−0.3 V vs Ag/AgCl	0.7	3.6	(Deeke et al., 2015)
Granules with intermittent contact to anode plate in integrated discharge cell via transport using internal gas lift (moving bed)	7700	2300	0–0.15 V vs Ag/AgCl with periods of controlled current	40.1	257	(Borsje et al., 2019)

The highest current, both based on reactor and granule volume, has been achieved with a single granule fixed to the current collector (Pt wire); it produced a current of 77 kA m^−3^ granule, several orders of magnitude higher than that produced in larger scale systems with intermittent contact with the current collector (Borsje et al., 2016). Both when the reactor and granule volumes increase, the volumetric currents decrease. This might be due to the poor connections between the different carbon granules and between the granules and the current collector. This also indicates that there is room for further improvement in the design of scaled-up systems. Proper contact between granules and current collector seems the most important design criterion for scaling up these reactors, as the current density produced by a single granule which is contacted via a wire (Borsje et al., 2016; Caizán-Juanarena et al., 2019) is several times higher than the current density produced by granules that are in contact with the current collector via fluidization (Table 1).

Many of the studies are fluidized bed systems using different fluidization methods, liquid or gas based, that cause the granules to be contacted with the current collector. The granules can be either charged and discharged in the same reactor (Borsje et al., 2019; Li et al., 2014; Liu et al., 2014a, Liu et al., 2014b; Tejedor-Sanz et al., 2017; Wang et al., 2014b) or can be charged in one reactor and discharged in another reactor (Deeke et al., 2015). The granules are transported between both reactors through recirculation. Tejedor-Sanz and co-workers (Tejedor-Sanz et al., 2017) showed that the system with one reactor, where the current collector was immersed in the fluidized bed, increased the performance compared to charging and discharging in two separate reactors, as used by the gas lift reactor developed by Deeke and co-workers (Deeke et al., 2015). The gas flow caused a circulation of the liquid flow that transported the granules past the current collector. To improve the discharge characteristics, a moving bed reactor has been designed in which the granules have longer contact time with the current collector (Borsje et al., 2019). This resulted in a higher current density compared to the fluidized bed system. The highest current density of a fluidized system was achieved by Liu and co-workers (Liu et al., 2014b) where the AC granules were fluidized and brought in contact with the current collector through stirring of the anolyte and granules. The authors identify that stirring requires energy and that a larger scale system needs a different fluidization method (Liu et al., 2014b). Further studies are needed to investigate which mode of fluidization is preferred to ensure the best contact between AC granules and current collector.

#### 4.1.2 Other materials for capacitive bioanodes

Besides activated carbon, other materials have been tested as capacitive anodes. For example, transition metal oxides (e.g. based on Fe, Co, Ni, Mn, Cu) show pseudocapacitive behaviour, although most have yet to be shown as capacitive bioanodes (Patake et al., 2009; Salunkhe et al., 2017). Wang and co-workers (Wang et al., 2016) successfully applied carbon felt coated with MnO_2_ as a capacitive bioanode. The specific capacitance of the coated felt was increased fivefold compared to the carbon felt. This led to an increase in both peak (30–40 times higher) and continuous current production (8.5–8.9 times higher) and showed the possibility to store charge (8.8–9.3 times higher). Liang and co-workers (Liang et al., 2017) investigated charge and discharge ratios of a reduced graphene oxide anode with MnO_2_ modification, which produced 16% higher current density when operated intermittently. However, after 11 h of operation, this was reduced to 10% higher current in intermittent mode. Another study showed that a MnO_2_/felt bioanode lost 88% of its capacitance when not protected by a conductive polymer layer (polymer/MnO_2_/felt). The polymer/MnO_2_/felt bioanode showed a higher electron transfer efficiency than the carbon felt or MnO_2_/felt bioanodes (Liu et al., 2018). This could be beneficial for high average current densities under intermittent operation mode (i.e. fast discharge of stored charge). Future studies still will have to show the benefits of this electrode in long term intermittent operation.

Also, other transition metal oxides have been tested as capacitive bioanodes. Ruthenium oxide (RuO_2_) has a high specific capacitance of over 800 F g^−1^ (Hong et al., 2014). A carbon cloth anode, modified with ruthenium oxide showed an improved current density by 21 times for a mixed community bioanode (4.2 A m^−2^) compared to an uncoated anode (0.2 A m^−2^) (Lv et al., 2012). Further study showed that similar to the MnO_2_ bioanodes described above, there was a 40% loss of capacitance over 6 months of intermittent operation (Lv et al., 2014). However, application of RuO_2_ and other noble metals involves high costs. Therefore, although these materials have been shown applicable as capacitive bioanodes, their use in larger scale systems is expected to be limited.

### 4.2 Integration of capacitive materials as cathodes

Besides being used as anode, capacitive materials can also be used as cathode with the aim to increase current and power densities of MFCs. Oxygen is the most used oxidant at the cathode due to its natural availability and high redox potential. Two types of cathodes are generally used in MFCs: i) fully submerged cathode and ii) gas-diffusion cathode. In the first case, the cathode is fully submerged in the catholyte liquid and operated with dissolved oxygen. The second case consists of a hydrophobic-type cathode structure that uses oxygen in the gas phase as electron acceptor.

Carbonaceous electrodes are attractive materials for cathodes (Santoro et al., 2017; Wang et al., 2014a; Yuan et al., 2016), as they are conductive, have low costs, and possess a high specific surface area. This high specific surface area has two advantages: (i) it can result in lower overpotentials for the oxygen reduction reaction, and (ii) it has capacitive properties that can offer advantages when the system is operated in intermittent mode. The cathode is positively charged due to redox environments (biological or chemical) and balanced out by ions (anions) naturally occurring in the wastewater. For the two types of cathodes used for oxygen reduction in MFCs, the gas-diffusion cathodes have a lower overpotential compared to submerged cathodes because of the higher oxygen concentration at the electrode surface. Whereas submerged cathodes have a large surface area exposed to the electrolyte solution, which is available for EDL formation, gas-diffusion electrodes are less exposed to the electrolyte and therefore, EDL formation is limited.

When capacitive materials are used as cathode, under open circuit conditions (charge), the cathode potential will increase to a maximum value (see Fig. 4). A high cathode potential gives a high cell voltage and results in high power density during discharge. The intermittent operation of a capacitive cathode can therefore be used to improve the energy and power output during discharge of a capacitive MFC. Activated carbon-based cathodes can be used in combination with catalysts, for example Fe-based materials (Fe-aminoantipyrine, Fe-AAPyr) (Kodali et al., 2017), and enzymes (bilirubin oxidase, BOx) (Santoro et al., 2016a). It was shown, using gas-diffusion cathodes, that the cathode open circuit potential (OCP) increased from +105 mV (AC) to +175 mV with Fe-AAPyr and up to +315 with BOx catalyst. Without catalyst, the maximum power was 0.67 mW (2.98 W m^−2^). Use of an Fe-AAPyr catalyst increased the power to 0.90 mW (4 W m^−2^), while the BOx catalyst increased the power to 1.47 mW (6.53 W m^−2^) (Santoro et al., 2015).

Another effect on integrating the advantages of capacitive materials and an improved ORR was addressed by Santoro and co-workers (Santoro et al., 2015). A gas-diffusion electrode was integrated with an additional electrode with high surface area (capacitance) in the electrolyte solution and short-circuited with the cathode electrode. The use of this additional electrode allowed the decrease of internal resistances by one order of magnitude and increased maximum power output with a factor of 10. The maximum power achieved with the additional capacitive electrode increased the maximum power of the gas-diffusion cathodes to 6 mW (26.7 Wm^−2^) for the AC cathode, 14 mW (62.2 Wm^−2^) for Fe-AAPyr cathode and 19 mW (84.4 Wm^−2^) for BOx cathode.

## 5 Applications of external capacitors for MFCs

As an alternative to the integrated use of capacitors in MFCs, capacitors can also be connected to MFCs through an external circuit. An external capacitor (of known capacitance) has the advantage of wider potential ranges at which charge/discharge cycles can occur compared to internal capacitors, as there is no living microorganism involved in the charge storage process. External capacitors of different sizes have been tested in both constant and intermittent modes, where especially the combination of external capacitors and intermittent operation mode has led to increased power outputs (Dewan et al., 2009, Dewan et al., 2010). This is similar to what was found with internal capacitors, where the electrodes were connected (on)/disconnected (off) to/from an external circuit. External capacitors can be adjusted to meet the desired power level and so match with the specific requirements of electronic devices. In fact, key electronic components such as capacitors, but also batteries, boost converters, inductors, transformers, diodes and other devices have been employed into different power management systems. The energy harvesting systems mainly used for MFCs with external capacitors are discussed below (Wang et al., 2015).

Successful examples of energy harvesting systems for MFCs based on intermittent energy harvesting have already been achieved, showing that the energy stored by external capacitors was greatly affected by the charge and discharge frequency or duty cycle (Ren et al., 2013). Charge-pump systems are another example for voltage boosting that use capacitors as energy storage devices. These systems (also called voltage multipliers) exploit the flow of current in a closed circuit to charge one capacitor and then discharge it into a second capacitor connected to the DC supply rail, which results in twice the voltage ‘seen’ at the load stage (Wang et al. 2015; Wang et al., 2012). Multiple capacitors can be used as accumulation stages to multiply the amount of source voltage to the desired amount. Pump-charge topologies are better suited for low current levels (<500 mA), due to the charge leakage characteristics of capacitors.

Boost converter-based solutions as energy harvesting systems for MFCs are another method reported in the literature (Wang et al., 2015). The key component is the DC/DC converter that is capable of boosting voltage to a higher value to power devices. In fact, this configuration is mainly used in sediment (or benthic) MFCs in which individual MFCs can only be connected in parallel and so boost the current at the same voltage, which would be insufficient for powering off-the-shelf sensors. This is due to the fact that MFCs are sharing the same electrolyte and therefore their connection in series is not possible. Sensors often require higher voltages to be powered and the currently available literature presents a range of diverse and successful examples of the utilization of this technique (Arias-Thode et al., 2017; Babauta et al., 2018; Donovan et al., 2008, Donovan et al., 2011, Donovan et al., 2013; Karra et al., 2014; Shantaram et al., 2005; Tender et al., 2008).

Maximum power point tracking (MPPT) systems have also been implemented in MFCs as energy harvesting methods (Alaraj et al., 2014; Degrenne et al., 2012; Do Park and Ren, 2012a, Do Park and Ren, 2012b). MPPT is dynamic and adapts to the changing MFC output and internal resistance due to environmental or physico-chemical perturbations (e.g. temperature, pH, substrate availability). The advantage of this system is the real-time tracking of the maximum power point and the energy harvesting at that specific point. Given that MFCs are dynamic, continuous optimisation through this technique enables better overall MFC performance.

The first examples of systems in which external capacitors or batteries were used as energy accumulators of MFCs have been Gastrobot (Wilkinson, 2000), the family of EcoBots (Ieropoulos et al., 2003, Ieropoulos et al., 2010; Melhuish et al., 2006) and, more recently, Row-bot (Philamore et al., 2015). The implementation of MFCs within robots led to a more compact and energetically autonomous system that does not require an external supply. In the example of Gastrobot, sugar was fed into a “stomach” populated with *E. coli*, whose (chemically) reduced digestate was fed into chemical fuel cells that extracted energy as electricity and used it to charge the batteries that were powering the robot (Wilkinson, 2000). The EcoBot-I and -II examples demonstrated that electrical energy could be recovered directly inside MFCs (i.e. the MFC stack was the digestive stage). This energy was transiently stored in electrolytic capacitors that were facilitating a charge/discharge duty cycle, which kept the phototactic robot moving towards the light in a pulsated manner (Ieropoulos et al., 2012). This was in order to demonstrate a “sleep/wake-up” pattern as part of an on-board energy management system, akin to animals in nature. EcoBot-III went beyond this level of operation since it incorporated a liquid circulatory system and was designed to move towards feeding and watering stations, in order to collect its own food and water; by ingesting (fresh food), digesting (collected food) and egesting (waste), it demonstrated autonomy through the completion of the thermodynamic cycle within a constrained environment. As for Row-bot, it was designed with a compliant lightweight embodiment so that it could operate on water. Inspired by the water boatman, and by rowing itself forward in a nutrient-rich water environment (akin to a polluted lake) whilst at the same time opening a “mouth”, Row-bot demonstrated the potential of ‘living’ in a polluted environment and utilizing the contaminated water as the feedstock for its on-board MFCs (Philamore et al., 2015).

In all three generations of EcoBot as well as Row-bot, external capacitors have been used for temporary storage of the harvested energy, which was only spent (capacitor discharge) when a pre-determined voltage threshold level was reached. From an energy management perspective, the use of capacitors allows the implementation of MFCs in applications where the level of power demanded by a system is greater than the instantaneous level of power produced. From a behavioural point of view, the use of capacitors allows the artificial agent to mimic life-forms, with periods of activity and dormancy that enable a more sustainable management of energy resources and reserves (Ieropoulos et al., 2012). This interaction between the living entities inside MFCs and the capacitors/electronic circuit artefacts has given rise to the notion of *artificial symbiosis* that forms part of Artificial Life and Living Machines.

## 6 Perspectives for research and application

To date, the power output of MFCs is limited. Therefore, the possibility of directly using the power output from MFCs for some practical applications remains a challenge, primarily due to the power requirements of state-of-the-art electronic devices. It will be therefore prudent to continue optimising MFCs for higher levels of performance, whilst at the same time invest in designing and developing electronic commercial products such as devices, motors and actuators that consume less instantaneous power. In this way, the gap that currently exists between off-the-shelf products' power requirements and MFC power output levels will be met from both directions. As described, capacitors can be used to shorten this gap when used in combination with MFCs, both internally and externally. The combination of MFCs and capacitors can: i) increase power production, ii) bring flexibility in the operation of the system, and iii) allow for scale-up of the system. Capacitive MFCs especially offer the advantage of increased power production when they are operated in intermittent mode, as a capacitor is only beneficial when the power requirement is short. Therefore, we envision its utilization mainly for pulsed applications such as sensors (e.g. temperature, pH, conductivity), lighting, movement of pumps or robots. Alternatively, capacitive MFCs can be operated as a fluidized bed reactor, where reactor operation (inflow, outflow) is continuous, but charging and discharging behaviour of the capacitive granules is intermittent due to the flow of granules past a discharge electrode (Borsje et al., 2019).

The cell voltage produced by a single MFC is typically lower than 1 V, which is the thermodynamic limit of the technology when operating with wastewater and using oxygen as electron acceptor. This means that, in order to meet the input requirements of electronic applications, either MFCs need to produce a high current (have a large surface area) so that they can be connected to amplifying/boosting electronics (Dewan et al., 2010, Dewan et al., 2014; Donovan et al., 2008, Donovan et al., 2011; Ewing et al., 2014; Shantaram et al., 2005) or multiple MFCs are connected as a stack in series, which may be sufficient to run the application directly (depending on input parameters) (Ieropoulos et al., 2008). Additionally, for practical application of MFCs, energy storage can be useful in order to reach a certain level of power output or to be used when power is required (pulsated or intermittent operation).

Several challenges remain before capacitive MFCs will be applied. To the best of our knowledge, two types of reactors have been proposed: i) fluidized reactors and ii) ceramic-based MFC stacks. By using inexpensive activated carbon granules, a new scaled-up fluidized reactor with 2 l volume was developed, in which the charging and discharging processes are separated (Deeke et al., 2015). This allows for a reduction of expensive materials like catalysts, as the discharging process takes place in a small part of the reactor. Based on performances of fluidized reactors in the literature, the first challenge is to ensure a good contact between the capacitive electrode and the current collector in order to minimize resistances and maximize current density. A second challenge is the competition between electrogens and other microorganisms, like methanogens, especially when real wastewater is used. Strategies to control the anode potential and substrate loading (Sleutels et al., 2016) should be further investigated to achieve high Coulombic efficiencies.

In parallel, a tentative of scaling up with a supercapacitive ceramic-based MFC stack of 1 l volume was pursued showing high levels of power generated under super-capacitive mode (Santoro et al., 2018, Santoro et al., 2019a). In these specific cases, carbon veil electrodes were used at the anode. Carbon veil electrodes are widely and successfully used as anodic material but do not possess the right properties for supercapacitive features. The cathodes were fabricated as activated carbon/polytetrafluoroethylene (AC/PTFE) mixture pressed over a stainless steel mesh (Santoro et al., 2018) and with the addition of Fe-based catalyst for enhancing the cathode potential (Santoro et al., 2019a). The cathode electrodes possess higher capacitive features that still could be improved even more. Material development and exploration will be certainly an area that requires more attention (Sharma et al., 2019). In a recent work using different MFC configuration, the anode capacitance has been increased significantly by embedding carbon veil with AC/PTFE layer (Santoro et al., 2019b). This might be a way to further pursue. Similarly, considering the cathode electrode, further improvements could be achieved in optimising the structure for enhancing the three-phase interface, as well as reducing the thickness, which consequently reduces the ohmic resistance and increase the overall output. An optimized design that could enhance the surface to volume ratio increasing the electrodes surface area in contact with the electrolyte should be envisioned in the prospective of higher current production.

Generally, the duration of charge/discharge cycles, named also as duty cycles, is an important challenge that requires further investigation. On the one hand, longer cycles result in higher cell voltages and more harvested charge. On the other hand, shorter cycles allow for a better use of the capacitive feature of the system (compared to the faradaic charge). In addition, the ratio between charging (or self-recharging) and discharging times need further study. Short charging times are attractive, as the time that the system is not producing electric power is limited, which will translate into a more efficient use of the capacitive material and eventually into smaller reactors (Caizán-Juanarena et al., 2019).

Finding a niche application or market for MFCs is of utmost importance. A clear example of this is sensing technology and robotics. Robotics has integrated this technology to reach self-sustained devices that could not have the same operation features otherwise. A large research field is foreseen on power management strategies, where a lot of research has been done in the past decades but their standardization, adaptation to the requirements of MFCs and their commercialization is still a weak spot.

The cost of capacitor materials is relatively low since the technology is based on high surface area carbonaceous materials (e.g. activated carbon, graphene) and/or transition metals oxides. Moreover, in order to enhance the voltage operational windows, ionic liquid or organic solvents are used as electrolyte. Electrolyte might be costly and not environmentally friendly but the low quantity utilized makes it only partially responsible for the cost. MFCs, by definition, need to be a low-cost technology in order to be competitive as power source and wastewater treatment system. Also in this case, carbonaceous materials with high electrical conductivity and potentially high surface area are used. The integration of capacitive materials on the electrodes of capacitive MFCs should not influence the cost, due to the low cost of high surface area conductive materials and oxides that are already commercially available.

Finally, this manuscript focused on the integration of capacitive electrodes in MFCs More generally, the use of capacitive electrodes could also offer advantages for other types of BESs. Whether capacitive features, both integrated in the electrodes but also externally, can also be used to boost other promising applications, like the CO_2_ reduction into valuable products in the cathode of MECs, remains an open research field.

## Declaration of Conpeting Interest

There are no conflicts of interest to declare.
